# Transnasal Endoscopic Resection of a Pituitary Metastasis From a Tonsillar Squamous Cell Carcinoma (SCC): A Case Report and a Review of the Literature

**DOI:** 10.7759/cureus.88146

**Published:** 2025-07-17

**Authors:** Matthias Matejka, Janos S Gellen, Theo Kraus, Christoph J. Griessenauer, Moritz F Ueberschaer

**Affiliations:** 1 Department of Neurosurgery, Chrisitan Doppler Clinic Salzburg, Paracelsus Private Medical University, Salzburg, AUT; 2 Department of Neuroradiology, Chrisitan Doppler Clinic Salzburg, Paracelsus Private Medical University, Salzburg, AUT; 3 Department of Pathology, Chrisitan Doppler Clinic Salzburg, Paracelsus Private Medical University, Salzburg, AUT

**Keywords:** computed tomography, pituitary insufficiency, pituitary metastasis, positron emission tomography, sella turcica, squamous cell carcinoma

## Abstract

This article presents the first documented case of a 61-year-old patient who underwent radical transnasal transsphenoidal endoscopic resection of a pituitary metastasis from a squamous cell carcinoma (SCC) of the tonsils. Only two other cases of pituitary metastases in SCC have been described in the literature to date.

The patient was diagnosed with p16 and human papillomavirus (HPV)-positive tonsillar SCC in June 2022. After initial radiotherapy and cisplatin-based chemotherapy, the patient had to undergo repeat surgeries due to local tumor recurrence. At that time, there was no evidence of metastatic disease. In August 2024, 26 months after the initial diagnosis, the patient presented to another institution with severe headaches, dizziness, nocturia, and pollakiuria. Laboratory tests revealed panhypopituitarism as well as diabetes insipidus. Magnetic resonance imaging (MRI) showed an intra- and suprasellar mass with increased F-fluoro-2-deoxy-D-glucose (FDG) uptake on positron emission tomography/computed tomography (PET/CT). A transcranial biopsy was performed that confirmed the diagnosis of a pituitary metastasis. Since radiotherapy carried a significant risk of impacting the unaffected optical system and the effect of chemotherapy was unknown, the interdisciplinary tumor board decided on resection. The patient underwent an extended endoscopic endonasal approach for radical tumor removal. The postoperative course was uneventful, and the patient received adjuvant radiochemotherapy and pituitary hormone replacement. Postoperative chemotherapy and radiation led to progression of the underlying disease, and the patient had to be switched to best supportive care six months after surgery.

This is the first case that reports the feasibility of a radical resection of a pituitary metastasis of SCC via the endoscopic endonasal approach. This paper reviews the risk factors, radiographic presentation, treatment, and follow-up of patients with pituitary metastases from oropharyngeal cancer.

## Introduction

Squamous cell carcinoma (SCC) of the tonsil is among the most prevalent forms of oropharyngeal cancer [1]. The tonsils are densely populated with lymphatic tissue, which predisposes them to metastasis to the cervical lymph nodes [2]. However, distant metastases of SCC can occur in 3-30% of cases [3]. The most common sites of metastasis from SCC are the liver, lungs, bones, and mediastinum [4].

Metastases from SCC to the central nervous system (CNS) are rare, occurring in only 1-2% of cases. This can be explained by a lower blood supply to the pituitary gland and thus a lower probability of hematogenous metastasis, a special metastasis pattern of squamous cell carcinomas, and by the anatomical barrier of the sella turcica. In general, metastases to the CNS tend to manifest in the later stages of the disease [5].

Headaches, visual disturbances, ophthalmological issues, hypopituitarism, and diabetes insipidus can all be indicative of pituitary metastases. The occurrence of diabetes insipidus or isolated ophthalmoplegia in patients with a history of malignancy should always raise suspicion of pituitary metastasis [5].

While metastases to the pituitary gland are not uncommon in breast or lung cancer, only two cases of metastasis to the pituitary gland in SCC have been reported [6-8]. Both of these patients underwent palliative radiochemotherapy. We now present the first case of a patient with pituitary metastasis from SCC that underwent successful radical tumor resection via an endonasal endoscopic approach.

## Case presentation

Initial diagnosis

A 61-year-old non-smoker initially presented with a globus sensation and increased symptomatology from his obstructive sleep apnea. In the initial examination, a mass was found in the right tonsil. A biopsy was performed to confirm the diagnosis, and an F-fluoro-2-deoxy-D-glucose positron emission tomography/computed tomography (FDG-PET/CT) was performed for staging. Histological examination revealed a p16-positive, human papillomavirus (HPV)-associated SCC with bilateral cervical lymph node metastases and no distant metastases (initial tumor stage cT3cN2cM0). Clinically, the patient presented with an Eastern Cooperative Oncology Group (ECOG) performance status of 0.

After a decision in the interdisciplinary tumor board, chemoradiation with cisplatin was recommended. Cisplatin was administered weekly in a total of five cycles. At the same time, the patient received radiation to the primary tumor and the affected lymph nodes in 31 individual doses of 2.25 Gray (Gy) each (total dose 69.75 Gy). Affected lymph drainage pathways and retropharyngeal lymph nodes on the right were irradiated with 31 individual doses of 2 Gy each (total dose 62 Gy).

Three months after completion of therapy, a staging FDG-PET/CT was performed again, showing a disease progression with increased accumulation in the left cervical lymph nodes. Consequently, a tonsillectomy and neck dissection were performed on the left side. Subsequently, a cutaneous local recurrence was observed at the site of the neck dissection scar, which could be completely resected under local anesthesia.

Metastatic progression

**Figure 1:**
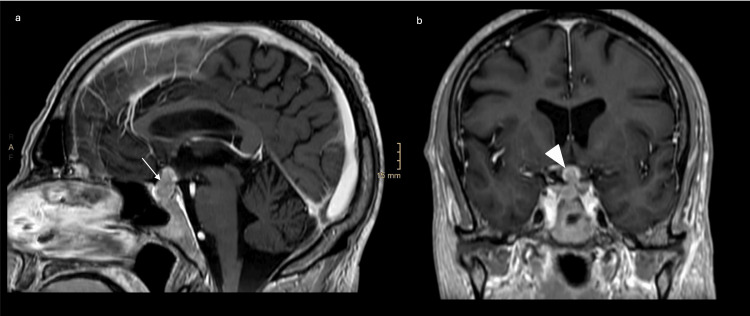
Initial cranial MRI with contrast medium (a) Sagittal T1-weighted MRI showing a contrast-enhancing mass in the pituitary fossa (arrow) and (b) a coronal T1-weighted MRI showing a contrast-enhancing mass (arrowhead) without infiltration of the cavernous sinus at the patient's initial presentation.

After one year without recurrence and constant clinical follow-up, the patient presented with loss of appetite, severe headaches, dizziness, massive nocturia, and pollakiuria. A magnetic resonance imaging (MRI) of the brain with contrast agent was performed, which showed a contrast-enhancing intra- and suprasellar mass measuring approximately 1.9 x 1.0 x 0.8 cm. The lesion appeared isointense on both T1- and T2-weighted images, with slightly irregular margins. Furthermore, there were two small contrast-enhancing metastases in both cerebellar hemispheres, too (Figure 1). In addition, FDG-PET/CT revealed a pathological FDG uptake in the sella turcica. Further accumulations were described in the left supraclavicular lymph nodes, mediastinal lymph nodes on both sides, in both hepatic lobes, and in the proximal right humerus. To confirm the diagnosis, the patient underwent a transcranial biopsy of the sellar mass at an outside institution. The histopathological examination confirmed a pituitary metastasis of the previously diagnosed p16-positive SCC.

**Figure 2:**
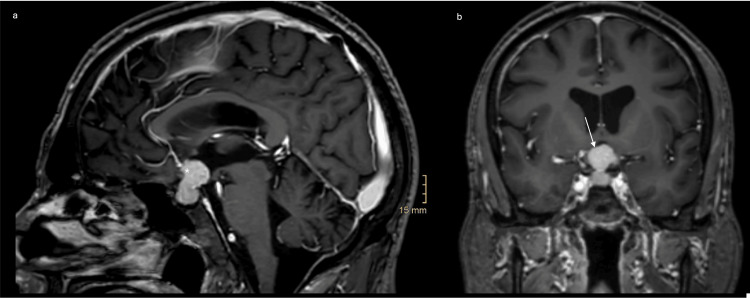
Preoperative cranial MRI with contrast medium Preoperative (a) sagittal and (b) coronal T1-weighted MRI showing a contrast-enhancing mass in the sella turcica with progressive suprasellar extension (asterisk/arrow) compared to the previous image.

Laboratory tests revealed panhypopituitarism (Table 1). In the acute situation, the corticotropic and thyrotropic insufficiencies were treated with hydrocortisone and thyroid hormone substitution. Central diabetes insipidus with a diuresis of up to eight liters per day was treated with desmopressin. The patient was transferred to the oncologist for further radiochemotherapy. Before the start of treatment, however, the pituitary metastasis showed significant suprasellar tumor progression into the third ventricle (Figure 2).

**Table 1:** Preoperative laboratory tests demonstrating the patient's hypopituitarism. The blood sample was taken under hydrocortisone and thyroid hormone substitution TSH - thyroid-stimulating hormone; FSH - follicle-stimulating hormone; LH - luteinizing hormone; ACTH - adrenocorticotropic hormone

Hormones	Results	Reference ranges
TSH	<0.01 mU/L	0.50-4.20 mU/L
fT4	1.21 ng/dl	0.93-1.70 ng/dl
fT3	1.7 pmol/L	3.1-6.8 pmol/L
FSH	<0.3 mU/L	1.5-12.4 mU/L
LH	<0.3 mU/L	1.7-8.6 mU/L
Prolactin	<2.0 µU/ml	86.0-324.0 µU/ml
Testosterone	<0.02 ng/ml	1.93-7.40 ng/ml
ACTH	<1.5 pg/ml	7.2-63.3 pg/ml
Cortisol	245 ng/ml	26.80-184.00 ng/ml
Growth hormone	<0.03 ng/ml	0.03-2.47 ng/ml
Insulin-like growth factor	52.2 ng/ml	59.2-189.0 ng/ml

The situation was discussed in an interdisciplinary neurooncological tumor board. The oncologists reported limited experience with the efficacy of chemotherapy for SCC metastases to the pituitary gland, and the radiotherapists perceived a significant risk of visual deterioration from radiotherapy due to the proximity of the metastases to the optic chiasm, despite an ophthalmologic examination that did not reveal any impairment of the patient's optic apparatus. Therefore, the patient was offered surgery.

Surgery

We discussed the two options of tumor resection via a transcranial versus a transnasal transsphenoidal approach. Since the trajectory of tumor growth was well suited for an endonasal approach and the patient suffered from panhypopituitarism, we recommended the endonasal approach, although it carries a significantly higher risk of postoperative cerebrospinal fluid fistula compared to the transcranial approach. This was a serious concern given the timely need for adjuvant radiotherapy. 

The patient underwent a transnasal, transethmoidal, transsphenoidal approach with resection of the middle turbinate on the right side and harvesting of a nasoseptal flap from the left side. After drilling the bone of the sella up to the tuberculum sellae, the dura of the pituitary fossa was incised, and the tumor-infiltrated gland was resected with grasping forceps, microscissors, and an ultrasonic aspirator. There was no evidence of cavernous sinus infiltration. Then, the suprasellar dura was opened, and the vessels underlying the optic chiasm were carefully dissected away from the tumor-infiltrated pituitary stalk. The stalk was then sharply resected. The tumor portion reaching into the third ventricle was reduced with an ultrasonic aspirator, and the lateral borders to the hypothalamus were dissected. After mobilizing the tumor at its margins, the residual tumor was removed with grasping forceps. The inspection with the 45-degree endoscope showed no residual tumor. For reconstruction, abdominal fat was placed into the suprasellar defect and covered with a synthetic dural inlay. Then the pituitary fossa was filled with abdominal fat, and another dural layer was positioned under the bone. Fibrin glue was used for fixation, and the nasoseptal flap was positioned over the reconstruction. Finally, a lumbar drainage was placed and left for three days postoperatively.

**Figure 3:**
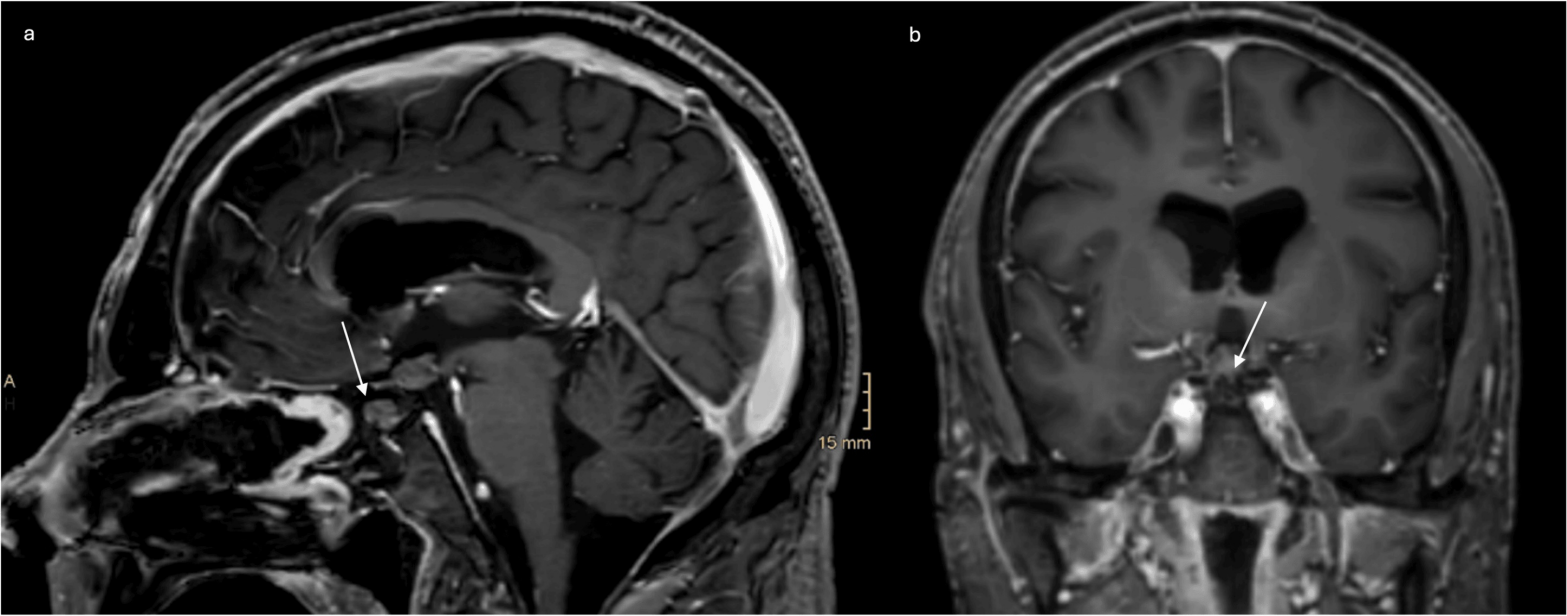
Postoperative cranial MRI with contrast medium Postoperative (a) sagittal and (b) coronal T1-weighted MRI showing complete removal of the metastasis. The arrows point at the abdominal fat graft that was used for the skull base reconstruction.

The postoperative MRI showed a complete resection without complications (Figure 3). The patient suffered from postoperative headaches, which could be satisfactorily treated with analgesics without signs of meningitis, hydrocephalus, or meningeosis carcinomatosa. The high diuresis volumes were controlled by increasing the dose of desmopressin. No electrolyte disturbances occurred, and the patient continued to receive hydrocortisone and thyroid hormone replacement. The ophthalmological examination reported normal vision and visual field after surgery. There was no gain in weight with respect to a potential hypothalamic injury.

Postoperative course

**Figure 4:**
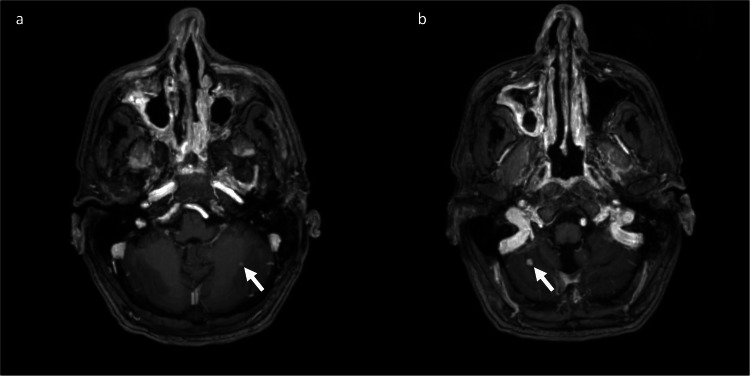
Postoperative cranial MRI with contrast medium (a) Axial T1-weighted MRI shows a contrast-enhancing mass in the left cerebellar hemisphere and (b) an axial T1-weighted MRI showing a contrast-enhancing mass in the right cerebellar hemisphere. Both masses are identical to the preoperative findings.

Eleven days after the surgery, chemotherapy with carboplatin, 5-fluorouracil, and pembrolizumab was started. Due to headaches that worsened during treatment, an MRI of the brain with contrast agent was repeated four weeks after the initial surgery. There were no signs of hydrocephalus or local tumor recurrence, with the small contrast-enhancing metastases in both cerebellar hemispheres remaining unchanged (Figure 4). A lumbar puncture was also performed, which ruled out meningeosis carcinomatosa. Consequently, whole-brain radiotherapy was planned for the patient as further treatment. In a follow-up MRI three months after the resection, there was still no evidence of local tumor recurrence. However, a restaging CT scan showed progression in the hepatic, osseous, pulmonary, and lymph node metastases. The chemotherapy was switched to paclitaxel and cetuximab, which showed further progression. The patient had to be switched to palliative care in February 2025.

**Figure 5:**
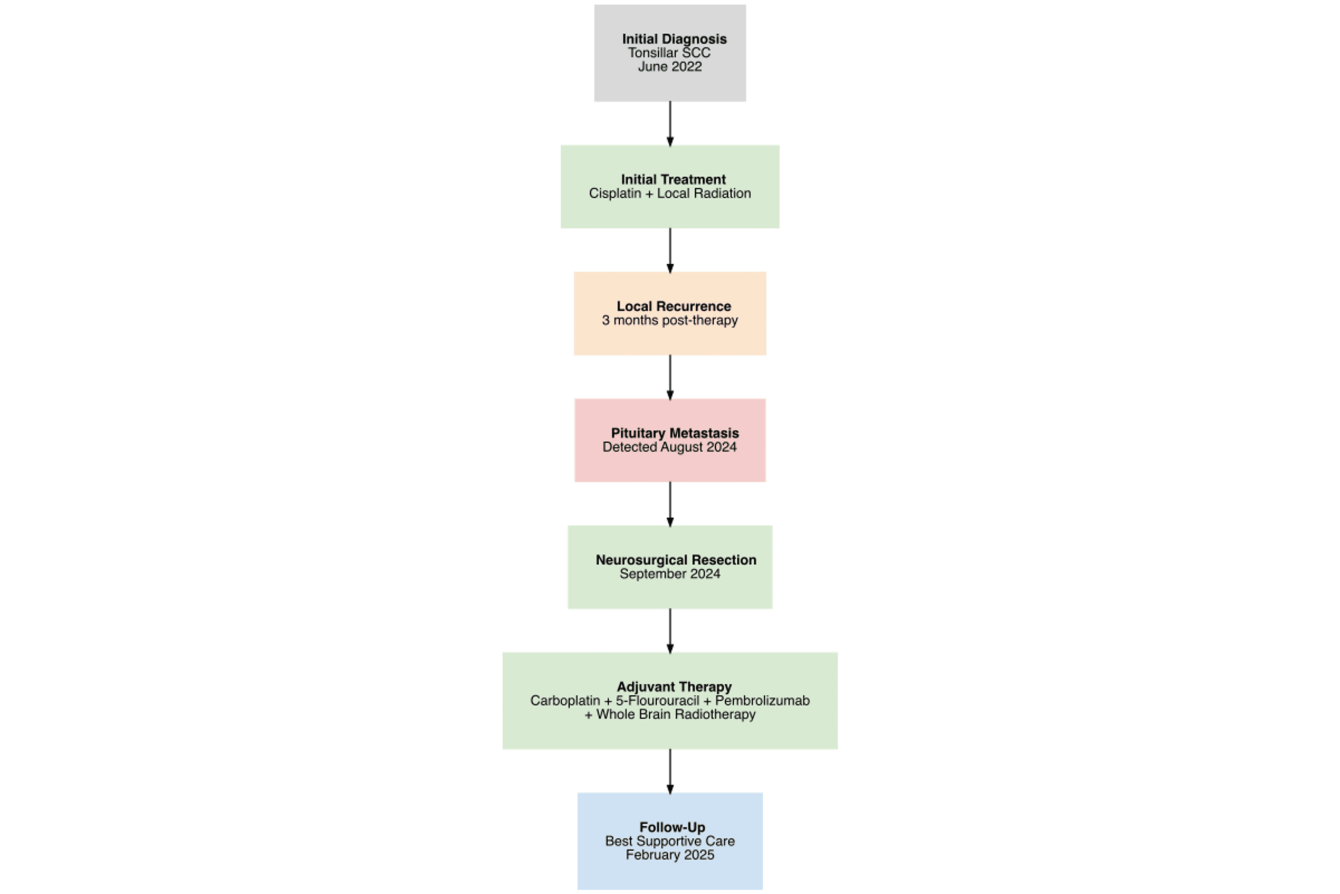
Patient's medical history The following is a concise synopsis of the patient's medical history, designed to facilitate enhanced comprehension SCC - squamous cell carcinoma

Figure 5 provides a concise synopsis of the patient's disease progression over time, thereby facilitating a comprehensive overview.

## Discussion

This is the first presentation of a patient with a pituitary fossa metastasis from an SCC that was resected transsphenoidally. To the best of our knowledge, only two similar cases have been published thus far (Table 2). The cases presented by Merchant et al. and Macdonald et al. described two male patients with a pituitary fossa metastasis from an SCC that were transsphenoidally biopsied. Merchant et al. report on a patient who presented two months after initial treatment with right oculomotor palsy and ipsilateral partial Horner syndrome (lid ptosis). Macdonald et al. presented a patient who presented with symptoms of syncope, vomiting, and fatigue after an initial treatment period of eleven months. Furthermore, the patient exhibited symptoms consistent with diabetes insipidus. In the report by Macdonald et al., hypopituitarism was observed in contrast to the case described by Merchant et al. In both cases, an MRI was performed, which showed a contrast-enhancing mass in the pituitary fossa. A transsphenoidal biopsy was performed in both cases to establish a definitive diagnosis. In both cases, the interdisciplinary tumor board reached a consensus on the administration of palliative radiotherapy [7,8].

**Table 2:** Reported cases of pituitary metastases of tonsillar squamous cell carcinoma The table summarizes the age, sex, initial tumor stage, histology, tumor type, treatment, pituitary function, and follow-up of previously published case reports by Merchant et al. and Macdonald et al. [7, 8].

Authors	Age (years)/ sex	Initial tumor stage	Histology	Type of surgery	Treatment	Pituitary function	Follow-up
Merchant et al. [7]	64/male	T3N0M0	p16 positive	Biopsy	Chemoradiotherapy	Normal	Palliative care
Macdonald et al. [8]	68/male	T4bN2M0	p16 positive	Biopsy	Chemoradiotherapy	Insufficiency	Not available

The presence of a pituitary fossa metastasis is indicated by a constellation of symptoms, including headache, double vision, visual field deficits, central diabetes insipidus, or symptoms of other hormone deficiencies [7,9,10]. In line with the report of Macdonald et al., the patient in this report presented with symptoms of panhypopituitarism [8]. 

The MRI of the brain presented a well-defined, solid mass emerging from the sellar region with distinctive suprasellar involvement, measuring approximately 1.9 x 1.0 x 0.8 cm. The lesion appeared isointense on both T1- and T2-weighted images, with slightly irregular margins. Post-contrast images showed avid enhancement of the lesional borders and a slight deviation of the optic chiasm similar to the case of Macdonald et al. [8]. The fluid attenuated inversion recovery (FLAIR) images showed edema involving the optic tracts and parts of the hypothalamus. The pituitary stalk and gland were entirely infiltrated by the tumor. There was no apparent invasion of the cavernous sinus or encasement of the internal carotid arteries. Preoperative CT imaging revealed no bony erosion, invasion of the sellar floor, or adjacent bony structures.

The management of SCCs is a topic of ongoing debate. In the early stages of the disease, surgical or radiation therapy alone is equally efficacious. In cases of advanced disease, a combination of surgery and postoperative radiotherapy is the preferred treatment modality [11]. However, there are currently no established treatment guidelines for patients with a pituitary metastasis from an SCC. A tissue sample of the pituitary mass is required for confirmation of the diagnosis [12].

The literature about radiation therapy for pituitary metastases is limited to retrospective analyses and case series. In a study by Chon et al., seven patients with pituitary metastases underwent hypofractionated radiation therapy. The primary endpoint was defined as one-year tumor control, which was 82.3% in this study [13]. In a subsequent retrospective study by Lin et al., 23 patients with pituitary metastases were subjected to irradiation. This resulted in a one-year local tumor control rate of 82.3% [14]. In a recent study, Wei et al. treated 18 patients with pituitary metastases using Gamma Knife radiosurgery, achieving local one-year tumor control of 94.4% [15]. In all cases described, high local tumor control was achieved. Chon et al. and Wei et al. achieved median survival times of 14 and 6.5 months, respectively [13,15]. In the study by Lin et al., the one-year survival rate was 72.9% [14].

In all three studies, the most prevalent primary tumors were non-small cell lung cancer and breast cancer, with no cases of pituitary metastasis of SCC [13-15]. In addition, potential sequelae of radiation in the context of pituitary metastases may encompass the development of hypopituitarism, optic neuropathy, and cerebrovascular incidents. In this case, the radiation therapists did not recommend radiation therapy due to its proximity to the optic apparatus. Therefore, the patient was offered transsphenoidal resection as an alternative treatment.

The transsphenoidal approach is preferred for most space-occupying lesions in the pituitary fossa, as it is less invasive and allows adequate visualization of the surgical site. In selected cases, such as substantial space-occupying lesions accompanied by intradural extension, invasion into the brain parenchyma, or encapsulation of neurovascular structures, a transcranial approach may be advantageous [16]. To date, there have been no studies that have directly compared these two approaches for the treatment of pituitary metastases.

In a retrospective analysis of 14 patients with pituitary metastases, Burkhardt et al. demonstrated that transsphenoidal resection is an effective method for tumor reduction, with a low complication rate and rapid symptomatic improvement in patients. A gross total resection was achieved in four patients [17].

A study by Zhao et al. demonstrated that the extended transsphenoidal approach in pituitary adenomas with intradural extension facilitates optimal visualization and high complete resection rates, accompanied by a low complication rate. These findings indicate that the extended transsphenoidal approach may be a viable and safe alternative for the removal of pituitary metastases that extend intradurally [18]. However, it should be noted that this approach may be associated with an increased rate of cerebrospinal fluid leakage [19]. According to the literature, the prevalence of this complication is reported at a rate of up to 21% [20]. In view of the systemic progression of the disease, this complication could delay urgent radio- and chemotherapies. Therefore, it is imperative to implement meticulous postoperative monitoring and ensure the implementation of adequate measures to prevent cerebrospinal fluid leakage [19,20]. If the tumor is partially intraventricular, as in the present case, care must also be taken to prevent tumor seeding, which was ruled out by the lumbar puncture. 

**Figure 6:**
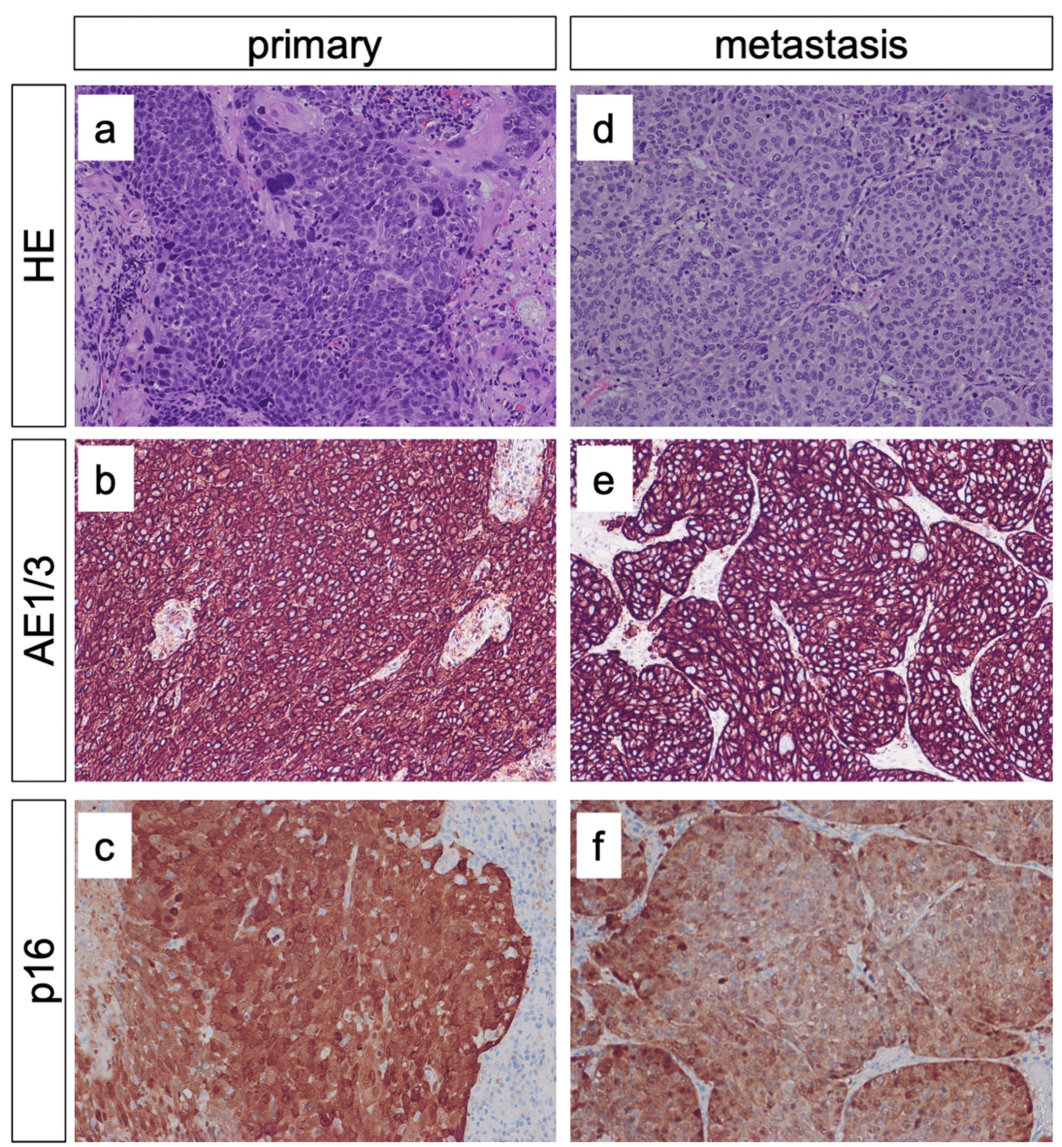
Histology of primary and metastatic carcinomas The primary carcinoma showed squamous cell differentiation (a) with expression of pan-cytokeratine (AE1/AE3) (b) and p16 protein (c). Metastases showed similar patterns with squamous cell differentiation (d), expression of pan-cytokeratine (AE1/3) (e) and p16 protein (f). In both the primary and the metastatic carcinomas, HPV 16 was detected.

In the histological examination after gross total resection, a metastasis of a p16-positive SCC was confirmed. As in the presented cases by Merchant et al. and Macdonald et al., p16 is a tumor suppressor protein that is often overexpressed in HPV-related oropharyngeal SCC [7,8,21]. Furthermore, an overexpression of p16 is associated with a significantly improved prognosis and a lower local recurrence rate, independent of the initial TNM classification and treatment modality [22,23]. The pituitary fossa mass and the primary tumor of the presented case were p16 and HPV positive and had similar histopathological patterns (Figure 6). It is estimated that approximately 44% of all HPV-related oropharyngeal carcinomas are tonsillar carcinomas, with 90% of these cases identified as SCCs arising from the mucosal lining of the oral cavity [24]. While SCCs are often associated with excessive smoking and drinking, HPV-related oropharyngeal carcinomas are not and tend to have a more favorable prognosis compared to smoking- and drinking-related oropharyngeal carcinomas [25,26].

In order to assess treatment response and exclude further distant metastases, an FDG-PET/CT is recommended as part of the follow-up protocol for oropharyngeal cancer. The negative predictive value of this examination is 91% if obtained within a six-month period following therapy [27,28]. However, the ability to detect brain metastases is limited. A prospective study conducted by Lee et al. demonstrated that an FDG-PET/CT only detects 24% of symptomatic and 19% of asymptomatic brain metastases [29]. The reason for this is the high physiological uptake of FDG by the whole brain, which can result in the presence of a metastatic lesion being masked until it is large enough to produce symptoms. At this juncture, it is possible that an invasion of surrounding structures, such as the cavernous sinus or the internal carotid artery, may have already occurred [30]. Therefore, MRI scans on a regular basis are important for follow-up examinations.

## Conclusions

This is the first case of a pituitary fossa metastasis of an SCC that was resected transsphenoidally. It is recommended that patients with SCC undergo prompt neuroimaging, particularly in the later stages of the disease when unusual new symptoms emerge, such as unilateral cranial nerve palsies or central diabetes insipidus. A biopsy is required for a definitive diagnosis to be made. Nevertheless, if radiation therapy is contraindicated due to the proximity of the lesion to the optic system, a transsphenoidal resection may represent a viable treatment option in selected cases. Finally, regular restaging and cranial imaging for follow-up are of immense importance.

## References

[1] Weatherspoon DJ, Chattopadhyay A, Boroumand S, Garcia I (2015). Oral cavity and oropharyngeal cancer incidence trends and disparities in the United States: 2000-2010. Cancer Epidemiol.

[2] de Bree R, Mehta DM, Snow GB, Quak JJ (2001). Intracranial metastases in patients with squamous cell carcinoma of the head and neck. Otolaryngol Head Neck Surg.

[3] Khan S, Anichini G, Mian A, Kareem H, Syed N, O'Neill K (2021). Tonsillar carcinoma spreading metastases to central nervous system: case report and literature review. J Neurol Surg Rep.

[4] Goodwin WJ (2001). Distant metastases from oropharyngeal cancer. ORL J Otorhinolaryngol Relat Spec.

[5] Dobelbower MC, Nabell L, Markert J, Carroll W, Said-Al-Naief N, Meredith R (2009). Cancer of the tonsil presenting as central nervous system metastasis: a case report. Head Neck.

[6] Altay T, Krisht KM, Couldwell WT (2012). Sellar and parasellar metastatic tumors. Int J Surg Oncol.

[7] Merchant H, Rye DS, Smith JA (2020). Isolated pituitary fossa metastasis from a primary tonsillar squamous cell carcinoma: case report. J Laryngol Otol.

[8] Macdonald AR, Ali AS (2023). Pituitary metastasis from tonsillar carcinoma presenting with hypopituitarism. Cancer Rep Rev.

[9] Al-Aridi R, El Sibai K, Fu P, Khan M, Selman WR, Arafah BM (2014). Clinical and biochemical characteristic features of metastatic cancer to the sella turcica: an analytical review. Pituitary.

[10] Patel KR, Zheng J, Tabar V, Cohen MA, Girotra M (2020). Extended survival after surgical resection for pituitary metastases: clinical features, management, and outcomes of metastatic disease to the Sella. Oncologist.

[11] Da Mosto MC, Zanetti F, Boscolo-Rizzo P (2009). Pattern of lymph node metastases in squamous cell carcinoma of the tonsil: implication for selective neck dissection. Oral Oncol.

[12] Goulart CR, Upadhyay S, Ditzel Filho LF, Beer-Furlan A, Carrau RL, Prevedello LM, Prevedello DM (2017). Newly diagnosed Sellar tumors in patients with cancer: a diagnostic challenge and management dilemma. World Neurosurg.

[13] Chon H, Yoon K, Kwon DH, Kim CJ, Kim MS, Cho YH (2017). Hypofractionated stereotactic radiosurgery for pituitary metastases. J Neurooncol.

[14] Lin YY, Wu HM, Yang HC (2023). Stereotactic radiosurgery for pituitary and cavernous sinus metastases. J Neurooncol.

[15] Wei Z, Yavan S, Deng H (2022). The role of stereotactic radiosurgery in the multidisciplinary management of pituitary metastases. Pituitary.

[16] Luzzi S, Giotta Lucifero A, Rabski J, Kadri PA, Al-Mefty O (2023). The party wall: Redefining the indications of transcranial approaches for giant pituitary adenomas in endoscopic era. Cancers (Basel).

[17] Burkhardt T, Henze M, Kluth LA, Westphal M, Schmidt NO, Flitsch J (2016). Surgical management of pituitary metastases. Pituitary.

[18] Zhao B, Wei YK, Li GL (2010). Extended transsphenoidal approach for pituitary adenomas invading the anterior cranial base, cavernous sinus, and clivus: a single-center experience with 126 consecutive cases. J Neurosurg.

[19] Baiano C, Somma T, Franca RA (2022). Evolution in endoscopic endonasal approach for the management of hypothalamic-pituitary region metastasis: a single-institution experience. Front Oncol.

[20] Dusick JR, Esposito F, Kelly DF, Cohan P, DeSalles A, Becker DP, Martin NA (2005). The extended direct endonasal transsphenoidal approach for nonadenomatous suprasellar tumors. J Neurosurg.

[21] Kida K, Terada T, Uwa N (2018). Relationship between p16 expression and prognosis in patients with oropharyngeal cancer undergoing surgery. In Vivo.

[22] Weinberger PM, Yu Z, Haffty BG (2004). Prognostic significance of p16 protein levels in oropharyngeal squamous cell cancer. Clin Cancer Res.

[23] Fischer CA, Zlobec I, Green E (2010). Is the improved prognosis of p16 positive oropharyngeal squamous cell carcinoma dependent of the treatment modality?. Int J Cancer.

[24] Hocking JS, Stein A, Conway EL, Regan D, Grulich A, Law M, Brotherton JM (2011). Head and neck cancer in Australia between 1982 and 2005 show increasing incidence of potentially HPV-associated oropharyngeal cancers. Br J Cancer.

[25] Maxwell JH, Kumar B, Feng FY (2010). Tobacco use in human papillomavirus-positive advanced oropharynx cancer patients related to increased risk of distant metastases and tumor recurrence. Clin Cancer Res.

[26] Fakhry C, Westra WH, Li S (2008). Improved survival of patients with human papillomavirus-positive head and neck squamous cell carcinoma in a prospective clinical trial. J Natl Cancer Inst.

[27] Castaldi P, Leccisotti L, Bussu F, Miccichè F, Rufini V (2013). Role of 18F-FDG PET-CT in head and neck squamous cell carcinoma. Acta Otorhinolaryngol Ital.

[28] Tantiwongkosi B, Yu F, Kanard A, Miller FR (2014). Role of (18)F-FDG PET/CT in pre and post treatment evaluation in head and neck carcinoma. World J Radiol.

[29] Lee HY, Lee KS, Kim BT (2009). Diagnostic efficacy of PET/CT plus brain MR imaging for detection of extrathoracic metastases in patients with lung adenocarcinoma. J Korean Med Sci.

[30] Galldiks N, Langen KJ, Albert NL (2019). PET imaging in patients with brain metastasis-report of the RANO/PET group. Neuro Oncol.

